# Restricting Periodontal Treatment Frequency: Impact on Tooth Loss in Danish Adults

**DOI:** 10.1111/cdoe.13022

**Published:** 2024-12-23

**Authors:** Eero Raittio, Rodrigo Lopez, Vibeke Baelum

**Affiliations:** ^1^ Department of Dentistry and Oral Health Aarhus University Aarhus Denmark; ^2^ Institute of Dentistry University of Eastern Finland Kuopio Finland; ^3^ Center for Translational Oral Research ‐ Periodontology, Department of Clinical Dentistry University of Bergen Bergen Norway; ^4^ School of Dentistry, Faculty of Medicine Pontificia Universidad Católica de Chile Santiago Chile

**Keywords:** Dental services research, Epidemiology, Periodontitis, Public health policy, Statistics

## Abstract

**Objective:**

The study aimed to estimate the effect of a periodontal treatment policy that would restrict the receipt of periodontal therapy to no more than once every second year, on the 10‐year risk of tooth extraction among Danish adults.

**Methods:**

Data from linked nationwide Danish registers consisted of a random sample of 20 000 50‐year‐olds who were followed from the beginning of 1990 to the end of 2021. The longitudinal modified treatment policies’ causal inference framework was used. In each of two slightly different counterfactual scenarios, the receipt of supragingival or subgingival periodontal therapy was restricted to no more than once every second year. The cumulative incidence of tooth extraction from 2012 to 2021 was compared between the counterfactual scenarios and the observed periodontal visiting pattern, while informative censoring, and time‐varying and time‐invariant confounding were accounted for using the social, economic and dental service utilisation history.

**Results:**

During the 10‐year follow‐up period, 5021 (25.1%) individuals received at least one tooth extraction. In the two counterfactual scenarios, the number of years receiving supragingival or subgingival periodontal therapy was 30%–50% lower than in the observed data. The 10‐year cumulative incidence of tooth loss was practically the same in the two counterfactual scenarios as under the observed periodontal visiting patterns.

**Conclusion:**

The findings indicate that a considerable decrease in the number and frequency of periodontal care visits would not have significant impact on the incidence of tooth loss in Denmark.

## 1 Introduction

The purpose of periodontal care is to preserve the long‐term stability of the tissues surrounding the teeth in periodontitis patients by integrating diagnostic (examinations, check‐ups), preventive (e.g., oral hygiene instructions) and therapeutic (e.g., professional tooth cleaning or subgingival scaling) measures. Similar diagnostic, preventive and therapeutic approaches are used to prevent the incidence of periodontitis [1, 2, 3]. Regular preventive and supportive periodontal care has since long been widely recommended, for example, by the European Federation of Periodontology and its regional representatives, and it is currently recommended that supportive periodontal care visits scheduled regularly based on the patient's individual needs every 3–12 months for every periodontitis case [4, 5]. Despite the absence of strong evidence from randomised controlled trials about the ideal timing or content for supportive periodontal [6, 7, 8, 9, 10, 11] or preventive dental visits, including professional scaling and polishing [1, 2], these frequent annual or even biannual dental visits have been adopted in many wealthy Western societies [2, 12, 13, 14, 15].

Under the current disease paradigm reflected by the 2017 Classification of Periodontal and Peri‐Implant Diseases and Conditions, co‐presented by the American Academy of Periodontology and the European Federation of Periodontology [16], the estimated prevalence of periodontitis in adults is extremely high, 70%–100% [17, 18, 19, 20, 21, 22]. Understandably, the prevention, treatment and control of periodontitis by professionals therefore pose a considerable economic burden for every society. For instance, it has been estimated that periodontal care expenses take up 0.61% of all annual health care spending in Denmark [12], and this estimate did not include prevention or treatment for the mildest, and most common periodontitis cases, which can be treated with supragingival scaling only. Much larger resources would have to be spent if the recommendations were, or could be, followed [18]. The situation calls for reconsideration with respect to more feasible, cheaper and still effective ways of treating and preventing periodontitis professionally worldwide.

In addition to other factors determining availability, accessibility and affordability of dental services, such as socioeconomic conditions and reimbursement policies, adherence to regular and frequent life‐long periodontal care depends on patients' and clinicians' values and preferences. Generally, regular and frequent dental visiting patterns are greatly appreciated by many patients and clinicians alike [6, 23, 24], although these practices are not universally followed within any society [12, 15, 25]. Since 2013, a national oral healthcare guideline has been in place in Denmark, which recommends that the frequency of dental visits and their contents be determined on the basis of an evaluation of dental and periodontal disease activity and risk factor modifiability, and recommends recall interval range between 12 and 24 months [26]. Hence, in 2013, 79% of Danish adults aged 45 and older reported having annual preventive dental visits [27], which involve a dental check‐up examination, typically coupled with one or more of the following services: general or individualised oral hygiene advice, fluoride application, and routine scale and polish. Other subsidised services include subgingival instrumentation and periodontal surgery, restorative (non‐prosthetic) treatments and endodontics, all of which can be added as needed, just as subsidised control‐of‐treatment visits are possible. It has thus been estimated that about 20%–24% of adult Danes attending dental care receive subgingival instrumentation or periodontal surgical services within a year [12]. Due to a rather generous and universal reimbursement policy in Denmark, the annual preventive dental visits are subsidised for the entire adult population, and their contents are recorded in a national administrative register. These widespread, yet nonuniversal, dental visiting patterns in the Danish society thereby make it possible to investigate effectiveness and cost‐effectiveness of annual or more frequent supportive or preventive periodontal care services by applying robust but flexible causal inference methodologies on these rich observational data with long follow‐up. These inference methodologies allow the definition and operationalisation of meaningful causal inference questions such as, ‘Would restricting or eliminating annual periodontal care visits change the 10‐year risk of tooth loss compared to the risk observed under the current dental visiting patterns?’ Answers to this kind of research question are essential to inform future resource allocation and reimbursement system development.

Such questions can be answered by utilising the longitudinal modified treatment policies framework [28, 29, 30]. Therefore, using this framework, the current study aimed to estimate the effect of a periodontal treatment policy that would restrict the receipt of subsidised periodontal therapy to no more than once every 2 year on the 10‐year risk of receiving at least one non‐surgical tooth extraction among Danish adults. As an alternative policy, the effect of a policy that would increase the probability of receiving subsidised periodontal therapy annually was investigated.

## 2 Methods

### 2.1 Data

The data used for the present analysis originate in a register‐based prospective dynamic cohort study established in Denmark. People were eligible for entry into the cohort at the time they reached the age of 20 years in the period from 1 January 1990 to 31 December 2021, if they were alive at the time of entry, and were permanent residents of Denmark. Information from the Civil Registration System, the Educational Register, the Income Statistics Register, the National Health Insurance Service Register and the Register for Selected Chronic Diseases was linked.

For the present analysis, persons born in 1960 who had been part of the cohort for the entire period between 1990 and 2011 (inclusive) were selected. Among them, a random sample of 20 000 persons was taken, representing 29% of the total eligible population born in 1960. The sample was thus uninterruptedly followed from 1990 to 2011, and from 2012 onwards until the earliest of the following events: death, permanent relocation abroad or the end of 2021. The rationale behind these criteria was to achieve a sample with a long follow‐up before the start of analytical period from the beginning of 2011 to the end of 2021. No attempts were made to define ‘non‐periodontitis’ cases for the purpose of excluding them, because it is likely that every dentate 50‐year‐old adult would meet the current periodontitis case defining criteria [17, 18, 19, 20, 21, 22].

The study was approved by the Danish Data Protection Agency (2015‐57‐0002) and Aarhus University (2016‐051‐000001‐914). In Denmark, large‐scale registry‐based studies like this do not require individual informed consent [31].

The study was reported according to the REporting of studies Conducted using Observational Routinely‐collected health Data (RECORD) statement [32].

### 2.2 Exposure

The exposure of interests was a three‐level categorical variable representing the receipt of periodontal therapy during the calendar year. Using data from the National Health Services Register, it was determined whether during the calendar year a person had received (1) subgingival or surgical periodontal treatments, (2) only supragingival treatments or (3) no periodontal treatment.

### 2.3 Outcome

The primary outcome was the occurrence of a non‐surgical tooth extraction, which was determined based on the treatment codes for non‐surgical tooth extractions in the National Health Services Register. This survival outcome was operationalised so that it changed from 0 to 1 in the year when the person first received one or more non‐surgical tooth extractions during the 10‐year analytical period. Surgical extractions, requiring gingival or mucosal incision, root sectioning or removal of bone tissue, were not considered because non‐surgical extractions are likely more specific for periodontitis‐related tooth loss. Reasons for extractions are not available via the National Health Services Register.

### 2.4 Covariates

As time‐invariant covariates, information about age, gender and origin (Danish/other); the highest educational attainment and municipality or region of residence were used. Additionally, ‘baseline’ summary measures of time‐varying covariates over 1990–2010 were formed: the total number of restorations received in the 21‐year period, the total number of non‐surgical or surgical extractions and six continuous variables representing the number of years with receipt of at least one of the following diagnostics and treatments: (1) supragingival treatment, (2) subgingival treatment, (3) surgical periodontal treatment, (4) endodontic treatment, (5) oral radiograph, (6) oral examination or (7) individual prevention registered to the National Health Services Register. Following the same logic, the sum of the individual's annual income percentile over 1990–2010 was calculated.

As annually time‐varying covariates from 2011 onwards, the study included income percentile and number of restorations within a calendar year, and four binary variables indicating whether the person had received (1) endodontic treatment, (2) oral radiograph, (3) oral examination or (4) individual prevention during the calendar year, and a binary variable that turned from 0 to 1 in the year the individual got diabetes mellitus type 1 or type 2 according to the Register for Selected Chronic Diseases and Severe Mental Disorders.

Missing data occurred only in the variable tallying the highest educational attainment, and a category representing unknown educational attainment (1.5% of observations) was thus formed. Details about the variables are provided in the Appendix S1.

### 2.5 Statistical Analyses

**FIGURE 1:**
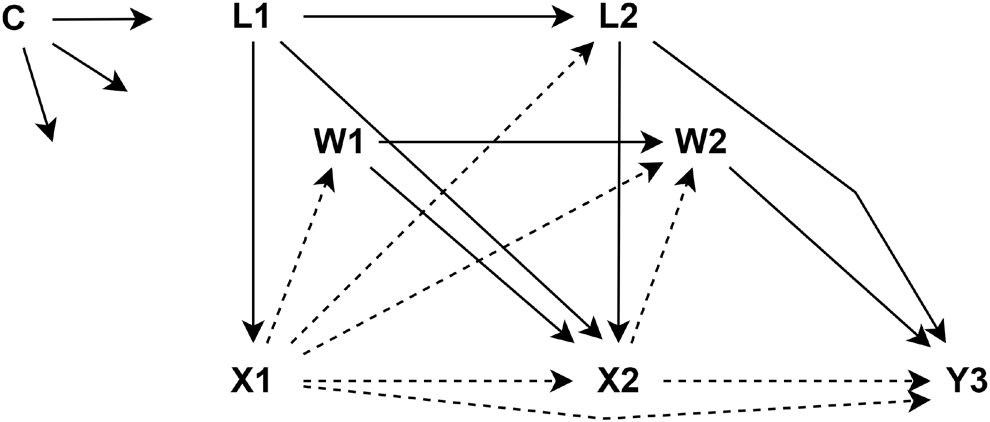
A simplified directed acyclic graph (DAG). C: baseline covariates, X: receipt of periodontal therapy, Y: tooth extraction, L: time‐varying covariates preceding the exposure each time point (income, diabetes) and W: time‐varying covariates not preceding the exposure each time point (restorations, examinations, radiographs, endodontics). Numbers refer to three timepoints (1–3). Please note that for the sake of clarity, not all assumed causal relationships are shown. It was thus assumed (but not shown) that L and W were also affecting Y, X, L and W with over‐year lags. In addition, C was assumed to affect all other nodes. Dashed lines indicate the causal paths of interest.

The data represent a complex longitudinal process with annual observations from 2011 to 2021. A simplified version of the data‐generation process for an outcome occurring at time 3 (year 2013) is shown in Figure 1. In addition to the baseline covariates (C), data comprise information on the participants' receipt of periodontal therapy (X), and tooth extractions (Y) as well as information on the time‐varying covariates (L and W). To ensure that receiving periodontal therapy always precedes the outcome, only outcome data from the last time point is used at each outcome timepoint 2012–2021, like Y3, 2013, in Figure 1. Within each year, we assume that some time‐varying covariates (L), income and diabetes status, causally influence the receipt of periodontal therapy, along with the baseline covariates (C) and the receipt of periodontal therapy in previous years (X1 → X2). The covariates representing time‐varying aspects of other dental treatments received (W) were assumed to act on X only with delay (a 1‐year lag at least, e.g., W1 → X2). The time‐varying covariates and the tooth loss outcome at each time point are assumed to be affected by the receipt of periodontal therapy in previous years, and so there is treatment‐induced (time‐dependent) confounding (e.g., L2 on the pathway X1 → L2 → X2 → Y3). This means that these variables are both confounders and mediators, and thus conditioning on them is both necessary and inappropriate for correctly estimating the treatment effect. Traditional statistical approaches are not suitable for robust causal inference in such cases, and thus methods accounting for time‐varying exposures and time‐dependent confounding are needed [28, 29, 30, 33, 34].

These methods include the longitudinal modified treatment policies framework [28, 29, 30], which has not yet been widely used in dental research but was recently employed to analyse the effect of increased tooth retention on the cognitive function among older Singaporean adults [35] and the effect of increased tooth retention on social participation among older Japanese [36]. Here, the longitudinal modified treatment policies framework was used to estimate the cumulative incidence of having at least one non‐surgical tooth extraction from 2012 to the end of 2021 in each of two different counterfactual scenarios, where the receipt of subsidised periodontal therapy (exposure) would be different from that actually observed (Observed):

1. A scenario where individuals could not receive any periodontal care in two consecutive years (Scenario 1).
2. A scenario where individuals could not receive supragingival care in two consecutive years but could receive subgingival or surgical periodontal care as actually observed (Scenario 2).

These scenarios were analysed using the R package ‘lmtp’ [28, 30], which is designed for studying the effects of time‐varying treatments and longitudinal modified treatment policies on survival outcomes in the presence of time‐dependent confounding (Appendix S2). In addition to the user‐defined modified longitudinal treatment policies (Scenarios 1 and 2), it allows us to estimate the cumulative incidence under the observed exposure and covariate history to serve as a reference (Observed).

Sequentially doubly robust estimation, which utilises both treatment and outcome regression models in a sequential manner, was used. Sequential double robustness means that if either the exposure or outcome model is correctly specified at any time point, the estimator remains consistent, ensuring unbiased estimates [28, 29, 30, 33].

The method also allows for the integration of a super learner, an ensemble of multiple regression and machine learning models, for the purpose of the estimation. These features are particularly advantageous when working with complex, high‐dimensional data for which it is challenging (impossible) to pre‐specify variable operationalisations (e.g., all relevant interactions) for standard inferential tools, such as a single generalised linear model [28, 29, 30].

Specifically, super learner ensembles, which include Bayesian generalised linear regression, LASSO regression and random forest models, were employed for our treatment and outcome models. In line with the assumed data generation process (Figure 1), these models included all baseline covariates and the entire available history of time‐varying covariates and treatment status separately for each 10 outcome timepoints. Similarly, informative censoring arising from loss to follow‐up due to death or relocation abroad before the end of the follow‐up period (2021) was adjusted for. To optimise and avoid overfitting in the super learner ensembles, two‐fold cross‐validation for the treatment models and 10‐fold cross‐validation for the outcome models were used [37]. Additionally, five‐fold cross‐fitting for the final estimators for the cumulative incidence were used [28, 29, 30]. Weights were checked for possible practical (observed) positivity violations (resulting in extremely high weights) and were found acceptable throughout the analysis period [29, 30]. Like in all causal inference, the untestable assumptions of consistency (individual's potential outcome under his or her observed exposure history is the outcome that will actually be observed for that person) [38] and exchangeability (no unmeasured confounding) were assumed to hold [28, 29, 30].

The cumulative incidence rates of having at least one non‐surgical tooth extraction in two counterfactual scenarios were compared to cumulative incidence rates under the observed periodontal therapy receipt patterns by plotting survival curves and in absolute terms using the risk difference and in relative terms using the risk ratio. Additional details about the models and their specifications are provided in the Appendix S2.

### 2.6 Alternative Analyses

The effect of an alternative policy of increased receipt of periodontal therapy on cumulative incidence of having at least one non‐surgical tooth extraction was also investigated. This policy was designed so that if a person had received periodontal therapy only in the first of two consecutive years, the exposure status in the later year was randomly replaced (50/50) with either observed exposure status (no periodontal therapy) or with the periodontal therapy received in the 1 year (Scenario 3). Otherwise, analyses were performed as described above.

## 3 Results

Of the 20 000 persons included in the cohort, 1173 (5.9%) died or moved permanently abroad during the follow‐up period from 2011 to 2021, and the data for this analytical period therefore included information over 188 270 person‐years. During the follow‐up, 5021 (25.1%) individuals had at least one non‐surgical tooth extraction. The sample characteristics in 2011 and 2020 in terms of time‐invariant and time‐varying covariates are shown in Table 1.

**TABLE 1:** The sample characteristics by baseline and time‐varying characteristics at the beginning and end of analytical period.

	2011	2020
	*n* (%), median (IQR)	*n* (%), median (IQR)
**Total** ***N***	20 000	18 827
**Baseline characteristics**		
Men	10 051 (50)	9344 (50)
Danish origin	18 954 (95)	17 859 (95)
Education		
Primary school	5126 (26)	4678 (25)
Vocational education	7903 (40)	7523 (40)
Short‐cycle higher education	1052 (5.3)	1017 (5.4)
Medium‐cycle higher education	3253 (16)	3121 (17)
Long‐cycle higher education	1216 (6.1)	1154 (6.1)
Other (4 levels)	1450 (7.3)	1334 (7.1)
Municipality/region		
North Jutland	1521 (7.6)	1437 (7.6)
Central Jutland	2798 (14)	2646 (14)
Southern Denmark	2756 (14)	2603 (14)
Capital region	3905 (20)	3687 (20)
Zealand	3235 (16)	3027 (16)
Other (10 diff. municipalities)	5785 (29)	5427 (29)
Sum of income percentiles 1990–2010	1384 (1085, 1674)	1397 (1103, 1680)
Sum of dental restorations 1990–2010	12 (6, 21)	12 (6, 21)
Sum of tooth extractions 1990–2011	1 (0, 2)	1 (0, 2)
Sum of years with endodontic treatment 1990–2010	1 (0, 2)	1 (0, 2)
Sum of years with oral examinations 1990–2010	11 (6, 12)	11 (6, 12)
Sum of years with individual prevention 1990–2010	1 (0, 2)	1 (0, 2)
Sum of years with oral radiographs 1990–2010	5 (3, 7)	5 (3, 7)
Sum of years with supragingival treatment 1990–2010	14 (7, 19)	14 (7, 19)
Sum of years with subgingival treatment 1990–2010	0 (0, 2)	0 (0, 2)
Sum of years with periodontal surgery 1990–2010	0 (0, 0)	0 (0, 0)
**Annually time‐varying characteristics**		
Income percentile	68 (48, 85)	65 (45, 83)
Diabetes type 1 or 2	767 (3.8)	1512 (8.0)
Number of restorations	0 (0, 1)	0 (0, 1)
Oral examination	13 490 (67)	11 700 (62)
Individual prevention	1588 (7.9)	4149 (22)
Endodontic treatment	1077 (5.4)	752 (4.0)
Oral radiograph	4431 (22)	3954 (21)

**FIGURE 2:**
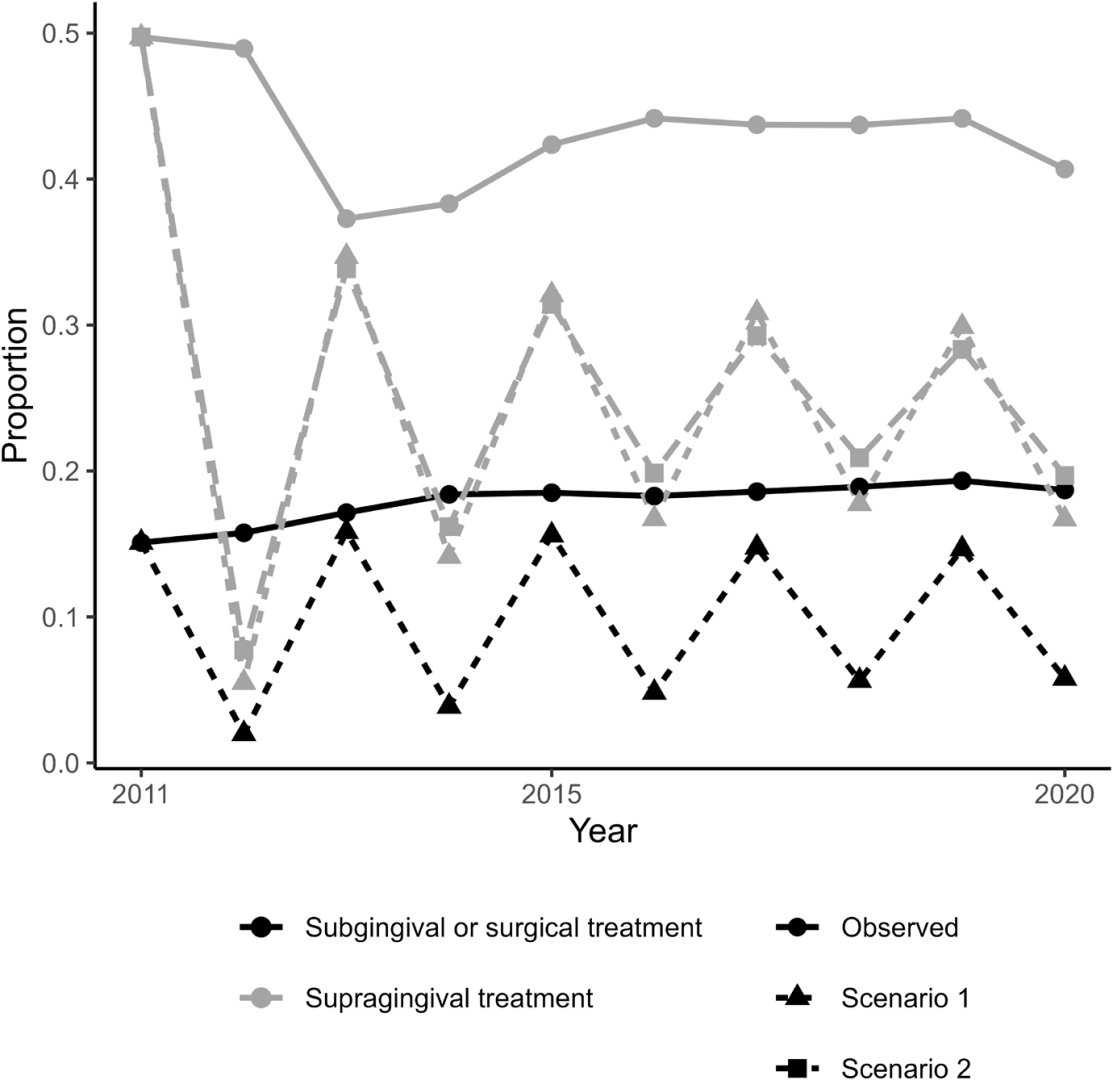
The proportions of people who received subgingival or surgical periodontal treatments or only supragingival treatment as observed between 2011 and 2020, and under the two counterfactual scenarios with modified periodontal treatment policy. Scenario 1: Individuals could not receive any periodontal care in two consecutive years. Scenario 2: Individuals could not receive supragingival care in two consecutive years but could receive subgingival or surgical periodontal care as actually observed. The curves for Scenario 2 and Observed are therefore identical for the receipt of subgingival or surgical periodontal care.

The observed average number of years with any periodontal therapy was 6.2 years (SD 3.6) corresponding to 117 256 person‐years over the 10‐year follow‐up period. In the counterfactual scenario where individuals could not receive any periodontal care in two consecutive years (Scenario 1), the average was 3.5 years (SD 1.8, 66 343 person‐years), and in the counterfactual scenario where individuals could not receive supragingival care in two consecutive years but could receive subgingival or surgical periodontal care as actually observed (Scenario 2), the average was 4.4 years (SD 2.8, 83 425 person‐years). Thus, these counterfactual scenarios roughly depict 50% and 30%, respectively, lower use of periodontal care over the 10‐year period (Figure 2).

**FIGURE 3:**
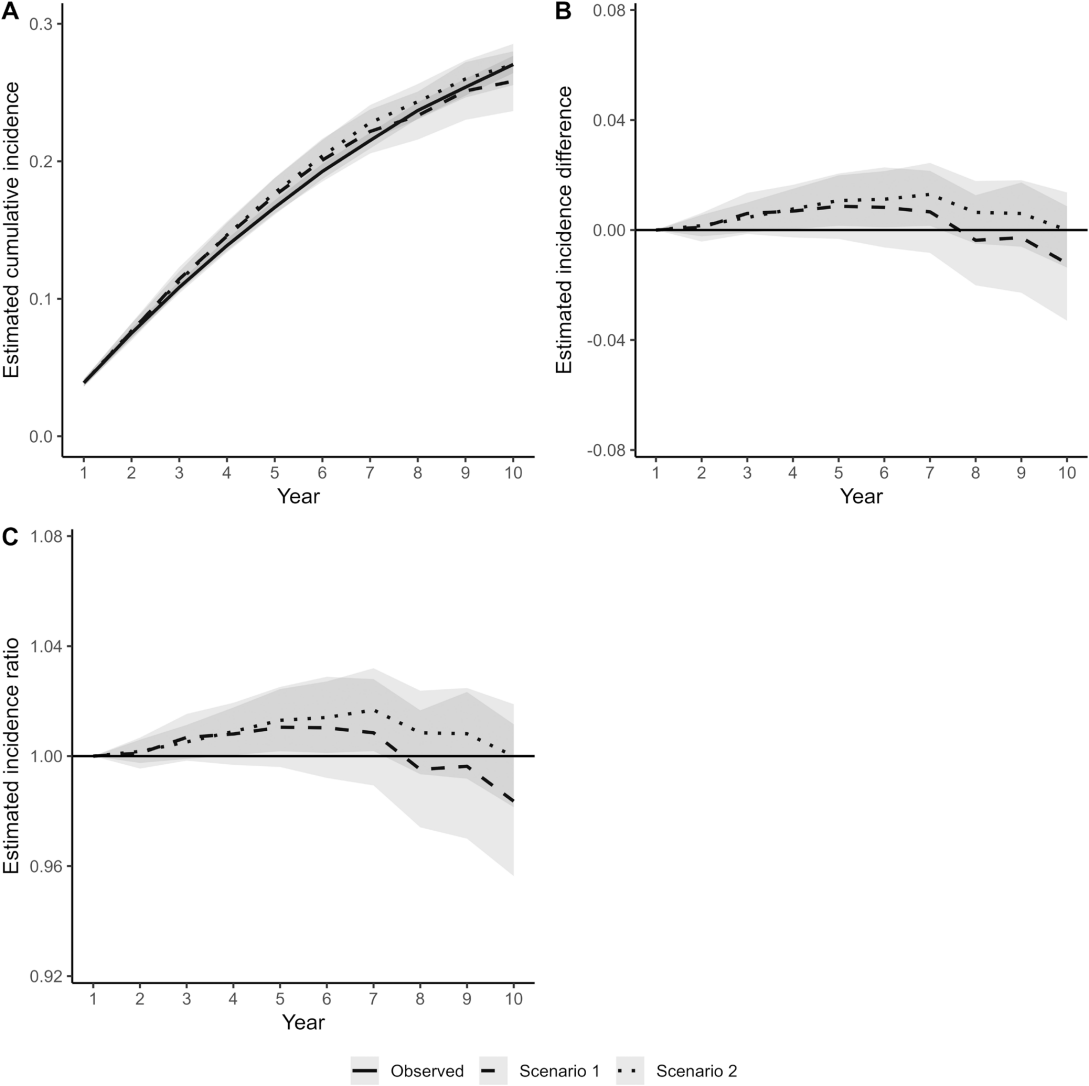
Differences in the estimated cumulative incidence of at least one non‐surgical tooth extraction between two modified treatment policies (Scenarios 1 and 2) and the observed periodontal treatment patterns in terms of cumulative incidence curves (A), incidence difference (B) and incidence ratio (C). The shaded areas represent 95% confidence intervals. Scenario 1: Individuals could not receive any periodontal care in two consecutive years. Scenario 2: Individuals could not receive supragingival care in two consecutive years but could receive subgingival or surgical periodontal care as actually observed.

The cumulative incidence of having at least one non‐surgical tooth extraction was slightly higher (1–2 percentage points) in the two counterfactual scenarios than seen under no modified treatment policy during years 3–7 after the start of the follow‐up (Figure 3 A,B). However, the cumulative incidences converged, and differences were minimal (1 percentage point or less) by the end of the 10‐year follow‐up.

**FIGURE 4:**
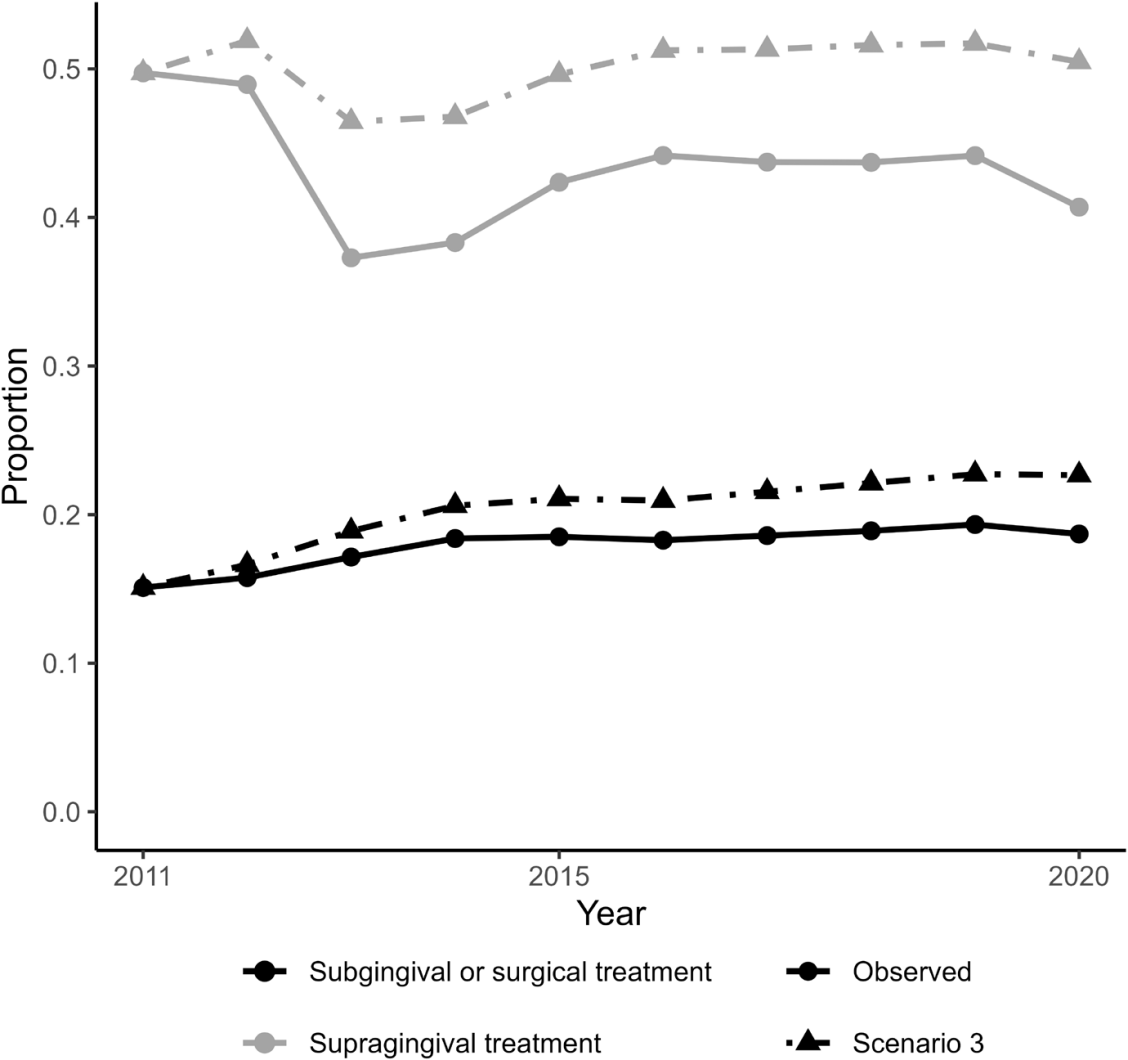
The proportions of people who received subgingival or surgical periodontal treatments or only supragingival treatment as observed between 2011 and 2020, and under the counterfactual scenario with modified periodontal treatment policy. Scenario 3: Individuals were more likely to receive periodontal care in consecutive years.

**FIGURE 5:**
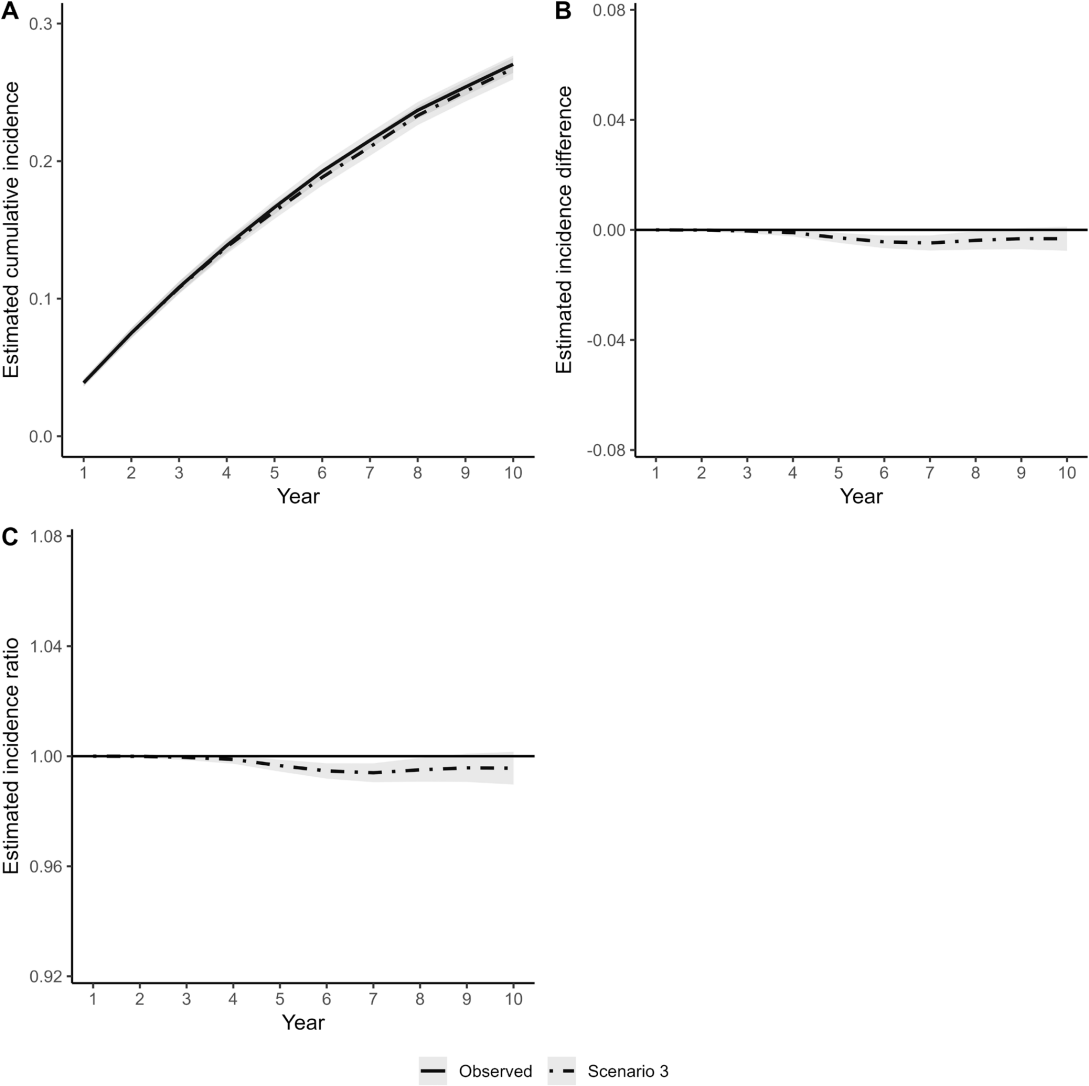
Differences in the estimated cumulative incidence of at least one non‐surgical tooth extraction between modified treatment policy (Scenario 3) and the observed periodontal treatment patterns in terms of cumulative incidence curves (A), incidence difference (B) and incidence ratio (C). The shaded areas represent 95% confidence intervals. Scenario 3: Individuals were more likely to receive periodontal care in consecutive years.

In the counterfactual scenario (Scenario 3) where the policy was to increase the annual periodontal care visits, the average number of years with any periodontal therapy rose from observed 6.2 (SD 3.6, 117 256 person‐years) to 7.1 (SD 3.7, 134 610 person‐years), corresponding to a 15% increase (Figure 4). In this scenario, the cumulative incidence of receiving at least one non‐surgical tooth extraction was minimally lower than in the observed data by the end of the 10‐year follow‐up (risk difference of −0.3%, 95% confidence interval: 0.1%; −0.8%, Figure 5).

## 4 Discussion

The current study analysed the impact of a policy limiting subsidised periodontal treatment to once every 2 years on the cumulative incidence of receiving at least one non‐surgical tooth extraction among 50‐year‐old Danish adults between 2011 and 2021. These hypothetical policies would lower the number of years receiving periodontal care by approximately 30%–50% in the population over the 10‐year follow‐up. Nonetheless, the findings showed that these policies would have minimal impact on the cumulative incidence of having at least one non‐surgical tooth extraction compared to observed periodontal treatment patterns. Furthermore, the additional analyses showed that a 15% increase in the use of periodontal care would not meaningfully decrease the 10‐year cumulative incidence of having at least one non‐surgical tooth extraction compared to the observed periodontal treatment patterns.

Despite using robust causal inference methods with diverse and nationally representative long‐term Danish data stemming from the universal reimbursement system and the use of a patient‐important outcome, the study has limitations. The data, primarily from administrative registers, lack specific information on the care provided, for example, its quality or the teeth involved. However, even though there may be some variations, it is likely that dental professionals rigorously adhere to the dental treatment code legal regulations in Denmark. Observational studies like the present are always susceptible to unmeasured confounding. Certain unaccounted factors related to tooth loss and receipt of periodontal therapy could have biased the estimates. Smoking, a major cause of periodontitis, is linked to increased risk of tooth loss and less frequent treatment, and may potentially bias the results towards protective effects [39]. If unmeasured aspects of periodontal and other oral diseases influence both treatment likelihood and tooth loss in the same direction, they may bias estimates towards no effect (or harmful effects) [39]. Finally, having non‐surgical tooth extractions was the only investigated outcome in this study, and it was not possible to investigate the effects on clinical outcomes, such as gingival inflammation, or on other patient‐important outcomes of periodontal care, such as orofacial function, appearance, pain or discomfort [39, 40, 41].

The findings of this study align with few randomised trials studying the impact of various intervals for dental check‐ups and periodontal treatment; more frequent visits are likely to offer little to no benefits for oral health [1, 2]. However, the periodontal community has placed more emphasis on observational studies [39, 42], which are often limited to single center [40, 43] or even individual practitioners [44]. These studies have consistently shown higher tooth loss rates among patients who did not adhere to recommended periodontal treatment [42]. However, these analyses are rarely adjusted for anything but time‐invariant confounders, if any adjustment has taken place. For example, a meta‐analysis [42] has suggested that individuals who adhere to regular periodontal therapy preserve about one tooth more every 8 years than do those with inconsistent adherence. Yet, this was based on unadjusted analyses, and thus assumed that those who adhere to care are exchangeable with those who do not adhere, which is an extremely unrealistic assumption. Once a while, researchers [45, 46], and also consultants commissioned by the periodontal community [47], have taken an alternative approach, and used simulations to investigate the (cost‐)effectiveness of periodontal therapy and of adhering to it. Unfortunately, these simulations seem to be based on overly optimistic assumptions about treatment effectiveness, for example, relying on the mentioned unadjusted meta‐analysis [42], or overly pessimistic assumptions on disease progression without treatment, for example, assuming progression as detected in Sri Lankan tea plantation workers, most of whom smoked, chewed betel nuts and never accessed dental care [48]. As a result, the present study offers a contrasting perspective on the (cost‐)effectiveness of regular or annual periodontal visits.

The current findings indicate that a considerable decrease in the amount and frequency of periodontal care visits would not have significant impact on the incidence of tooth loss in Denmark. It thus seems that currently in Denmark there are many people receiving regular periodontal care who could reduce their visiting frequency without considerable harm in terms of tooth loss incidence. In other words, the proportion of people benefitting from the frequent periodontal visiting patterns is likely much smaller than anticipated, for example, by the European Federation of Periodontology [4]. From a societal point of view, these findings support the aims of several public authorities in prioritising people with more severe oral diseases or higher risk of disease over those with better oral health by modifying subsidisation and regulation for dental visiting patterns in Denmark [12, 49] and elsewhere [23, 24, 50]. For children and adolescents, dental recall intervals have already been individualised and extended in the Nordic countries [51, 52, 53], just as the recommended recall intervals for adults have been extended based on patient risk profiles [26, 54]. To inform better resource allocation, future research could aim to determine the optimal periodontal care and the better dental check‐up patterns according to disease severity and risk factors, to identify more accurately those people benefiting most from periodontal care and dental check‐ups.

## 5 Conclusion

Using the longitudinal modified treatment policies framework, the current study indicates that a hypothetical policy that would restrict the receipt of periodontal therapy to no more than once every 2 year would have practically no effect on the 10‐year risk of receiving at least one non‐surgical tooth extraction among 50‐year‐old Danish adults. Likewise, it was shown that a considerable increase in the annual receipt of periodontal care would have a negligible effect on the 10‐year cumulative incidence of tooth extractions.

## Author Contributions

Eero Raittio: conceptualisation, data curation, investigation, writing – original draft, visualisation. Rodrigo Lopez: conceptualisation, writing – review and editing, funding acquisition, project administration. Vibeke Baelum: conceptualisation, data curation, writing – review and editing, project administration, supervision.

## Ethics Statement

The study was approved by the Danish Data Protection Agency (2015‐57‐0002) and Aarhus University (2016‐051‐000001‐914).

## Consent

The authors have nothing to report.

## Conflicts of Interest

The authors declare no conflicts of interest.

## Supporting information


Appendix S1.

Appendix S2.

## Data Availability

The data that support the findings of this study are available from Statistics Denmark. Restrictions apply to the availability of these data, which were used under licence for this study.
