# Supplementary material for: Restricting Periodontal Treatment Frequency: Impact on Tooth Loss in Danish Adults
Source: Community Dent Oral Epidemiol. 2024 Dec 23;53(2):205–15. doi: 10.1111/cdoe.13022 (PMC11892545; doi:10.1111/cdoe.13022)
Supplement: Supplementary file 2 — Appendix S2. [file CDOE-53-205-s001.docx]

**Supplementary information**

**Variables**

**Gender**: Men; Women. Information from: The Danish Civil Registration System (Pedersen 2011). Variable name in the register: KOEN.

**Origin**: Categorized into two groups: 1) Immigrants or descendants, and 2) persons of Danish origin. (Pedersen 2011). Variable name in the register: IE_TYPE

**Region/Municipality of residence**: Municipality if a person lived in one of ten biggest municipalities (Copenhagen, Aarhus, Odense, Aalborg, Vejle, Kolding, Esbjerg, Viborg, Randers, Frederiksberg) and one of the regions otherwise. Information from: The Danish Civil Registration System (Pedersen 2011). Variable names in the register: KOM and REG.

**Highest completed education**: categorized into eight groups:

- Primary school
- High school
- Vocational
- Short-cycle higher education or qualifying exam
- Medium-cycle higher education
- Bachelor
- Long cycle higher education
- Researcher education
- Unknown

Information from the Danish Educational Register (Jensen and Rasmussen 2011, Pallesen et al 2010). Categorized based on the variable in the register AUDD. Qualifying exam and Researcher education categories were combined with closest neighboring category due to their small size.

**Income percentile**: People were divided into 100 groups (percentiles) for each year from 1990 to 2021 based on their total annual personal income. Personal income in total is equal to the sum of business income, transfer income, property income (excluding calculated rental value of own home) and other non-classifiable income that can be attributed directly to the individual. The amount is before tax deduction, labor market contribution and special pension contribution, and interest expenses are not deducted. Information from the Danish Income Statistics Register (Baadsgaard and Quitzau 2011). Variable name in the register: PERINDKIALT_13.

**Dental service variables**

From the National Health Insurance Service Register (Andersen et al. 2011), we used the dental treatment codes that have been used over time since 1990 in the National Health Insurance scheme covering the subsidized dental care for all adult permanent residents in Denmark. According to the descriptions of the codes, services were categorized as follows:

| **Service** | **Codes** | **Details** |
| --- | --- | --- |
| Supragingival treatment | 1120, 1130, 2120, 1301, 1302, 1300 | Supragingival periodontal treatment (scale and polish) |
| Subgingival treatment | 1420, 1425, 1430, 1452, 1453, 1431 | Subgingival periodontal treatment (instrumentation) and control |
| Periodontal surgery | 1440, 1454 | Periodontal surgery and control |
| Dental restorations | 1501, 1502, 1503, 1504, 1505, 1506, 1507, 1509, 1551, 1552, 1553, 1554, 1555, 1556, 1557, 1558, 1559 | Amalgam and composite restorations |
| Oral examination | 1110, 1111, 1112, 1113, 1114, 1115, 1116, 1140, 1141, 1160, 1170, 1171, 1180, 2110, 2910, 1415 | Focused (e.g., periodontal) or general examinations and dental check-ups, includes also general oral hygiene advice |
| Individual prevention | 2920, 2930 | Individualized oral hygiene advice, fluoride application, smoking advice, or dietary advice |
| Oral radiograph | 1150, 1151, 1152, 1153, 1201, 1300 | Intraoral periapical or bitewing radiographs |
| Tooth extraction (non-surgical) | 1701, 1702, 1703, 1704, 1705 | Extractions without gingival or mucosal incision, root sectioning or removal of bone tissue |
| Surgical extraction | 1801 | Extractions with gingival or mucosal incision, root sectioning or removal of bone tissue |
| Endodontic treatment | 1600, 1601, 1602, 1603, 1604, 1605, 1606 | Root canal treatments, periapical surgery, and pulpotomies |

**Incident diabetes mellitus type 1 or 2:** Data on incident diabetes between 1997 and 2021 comes from the Registry for Selected Chronic Diseases and Severe Mental Disorders (The Danish Health Data Authority 2024). It combines information from the Danish National Prescription Registry (Pottegård A, et al. 2017), including all prescriptions in Denmark, and the Danish National Patient Registry (Schmidt M, et al. 2015) which contains information on all visits, procedures, and admissions to all Danish somatic hospitals, emergency departments, and hospital-associated outpatient clinics.

Individuals were classified as having incident diabetes mellitus (type 1 or 2) in a calendar year when one of the following criteria were met.

Diabetes type 1:

- Individuals registered with at least two purchases of insulin or insulin analogs (A10A, except combination medicines including GLP1-analogues and insulins, A10AE54 or A10AE56) in the Danish National Prescription Registry.
- Individuals registered with a relevant primary or secondary diagnosis (E10, diabetes type I, or its sub-codes under ICD-10) in the Danish National Patient Registry.

Diabetes type 2:

- Individuals registered with at least two purchases of medication aimed at lowering blood glucose (A10B, except A10BJ, A10BK01, A10BK03) or combination medicines including GLP1-analogues and insulins (A10AE54 or A10AE56) in the Danish National Prescription Registry.
- Individuals registered with a relevant primary or secondary diagnosis (E11, diabetes type 2, or its sub-codes under ICD-10) in the Danish National Patient Registry.

Excluded were:
Women who have been exclusively treated with metformin (ATC code A10BA02) and there were signs that they could have polycystic ovary syndrome (prescription for G03GB02, G03HB or diagnosis code E282).

Women who have a code for gestational diabetes (ICD-10 code O24.4) and who have only registered purchase of antidiabetics (A10) within 280 days before first contact or 280 days after last contact with gestational diabetes according to the Danish National Patient Registry.

**Methods**

**Modified treatment policies**

Modified treatment policies were constructed by editing the observed exposure status to reflect the hypothetical policies in the counterfactual scenarios. Specifically, these treatment policies were operationalized as shown below.

To recall, we have an exposure variable, which had three (ordered) levels: 0) no periodontal treatment, 1) only supragingival treatments; 2) subgingival or surgical periodontal treatments.

Scenario 1: Individuals could not receive any form of periodontal care in two consecutive years

From the second year onwards (2012), a person’s exposure status was set to 0 if their exposure status was either 1 or 2 in the previous year. The same approach was then used for the following years (2013-2020); such that the receipt of periodontal care in the previous year was determined by the modified exposure status, not the original exposure status. Therefore, for instance, for a person whose original exposure status was 2, 2, 1, 1 in four consecutive years (e.g. from 2011 to 2014), would be assigned a modified exposure status was 2, 0, 1, 0 in those four years, and for a person whose original exposure status was 2, 2, 0, 0, the modified exposure status would be 2, 0, 0, 0 in four consecutive years.

In terms of R script, this was implemented as follows:

for (year in 2012:2020) {

curr = paste0(“exposure_”, year) # exposure in current year

prev = paste0(“exposure_”, year - 1) # exposure in previous year

# Set current exposure to 0 if previous exposure was not 0 and current is not NA (lost to follow-up)

data[[curr]][!is.na(data[[curr]]) & data[[prev]] != 0] = 0

}

Scenario 2: Individuals could not receive supragingival care in two consecutive years but could receive subgingival or surgical periodontal care as actually observed

From the second year onwards (2012), if a person’s exposure status was 1 in two consecutive years, the latter exposure status was set to 0. The same approach was then used for the following years (2013-2020). Again, the receipt of periodontal care in the previous year was determined by the modified exposure status not the original exposure status. Therefore, for instance, for a person whose original exposure status was 1, 1, 1, 1 in four consecutive years, their modified exposure status was 1, 0, 1, 0.

In R, the data with observed exposure patterns was thus edited as follows:

for (year in 2012:2020) {

curr = paste0(“exposure_”, year) # exposure in current year

prev = paste0(“exposure_”, year - 1) # exposure in previous year

# Set current exposure to 0 if previous exposure was 1 and current is not 2 or NA (lost to follow-up)

data[[curr]][!is.na(data[[curr]]) & data[[prev]] == 1 & data[[curr]] != 2 ] = 0

}

Scenario 3 (alternative analysis): Individuals had higher probability of receiving periodontal care annually

If a person had received periodontal therapy only in the first of two consecutive years, the exposure status in the second year was randomly (50/50) replaced with the periodontal therapy received in the first year (1 or 2). Again, this was applied from the second year onwards (2012) and the same approach was used for the following years (2013-2020), and the receipt of periodontal care in the previous year was determined by the modified exposure status not the original exposure status. Therefore, for instance, for a person whose original exposure status was 1, 0, 0, 2 in four consecutive years, the modified exposure status could be either: a) 1, 0, 0, 2; b) 1, 1, 0, 2; or c) 1, 1, 1, 2.

In R, the data with observed exposure patterns was thus edited as follows:

for (year in 2012:2020) {

curr = paste0(“exposure_”, year) # exposure in current year

prev = paste0(“exposure_”, year - 1) # exposure in previous year

# Set current exposure randomly to 1 or 0 if previous exposure was 1 and current is 0 and set current exposure randomly to 2 or 0 if previous exposure was 2 and current is 0

data[[curr]] =

ifelse(data[[prev]] == 1 & data[[curr]] == 0, sample(c(1,0), length(data[[curr]]), replace=T),

ifelse(data[[prev]] == 2 & data[[curr]] == 0, sample(c(2,0), length(data[[curr]]), replace=T), data[[curr]] )

}

**Analyses**

Following the guidance and analysis script (github.com/kathoffman/lmtp-tutorial) as well as the earlier introductory article (Hoffman, et al 2024), the lmtp_sdr function was used to estimate the cumulative incidence of receiving at least one tooth extraction under the observed dataset and under the three counterfactual scenarios separately for each of the ten outcome timepoints (2012-2021) using the sequentially doubly robust (SDR) estimator. The datasets representing the three counterfactual scenarios with altered exposure patterns were provided with the “shifted” argument.

Super learner ensembles for outcome and treatment model included Bayesian generalized linear regression (SL.bayesglm), LASSO regression (SL.glmnet), and random forest models (SL.ranger) with default settings. While the outcome super learner ensemble also includes a simple mean model (SL.mean), this is not suitable for categorical variables such as the exposure/treatment variable in our case.

In line with guidance (Phillips, et al 2023) and to keep analyses computationally feasible, the number of cross-validation runs was lower in the treatment model (2-fold) than in the outcome model (10-fold). This was possible due to the large sample size and a sufficient number of observations at all three exposure levels (Phillips, et al 2023).

Finally, the lmtp_contrast function was used to calculate the risk differences and the risk ratios and their 95% confidence intervals for each time point. Results were visualized using ggplot2 package (Wickham 2016).

**References**

Andersen JS, Olivarius Nde F, Krasnik A. The Danish National Health Service Register. Scand J Public Health. 2011;39(7 Suppl):34-7

Baadsgaard M, Quitzau J. Danish registers on personal income and transfer payments. Scand J Public Health. 2011;39(7 Suppl):103-5.

The Danish Health Data Authority. Registry for Selected Chronic Diseases and Severe Mental Disorders [in Danish]. 2023. https://www.esundhed.dk/Emner/Operationer-og-diagnoser/Udvalgte-kroniske-sygdomme-og-svaere-psykiske-lidelser

Hoffman KL, Salazar-Barreto D, Williams NT, Rudolph KE, Díaz I. Studying continuous, time-varying, and/or complex exposures using longitudinal modified treatment policies. Epidemiology. 2024;35(5):667-675.

Jensen VM, Rasmussen AW. Danish Education Registers. Scand J Public Health. 2011;39(7 Suppl):91-4.

Pallesen PB, Tverborgvik T, Rasmussen HB, Lynge E. Data on education: from population statistics to epidemiological research. Scand J Public Health. 2010;38(2):177-83.

Pedersen CB. The Danish Civil Registration System. Scand J Public Health. 2011;39(7 Suppl):22-5.

Phillips RV, van der Laan MJ, Lee H, Gruber S. Practical considerations for specifying a super learner. Int J Epidemiol. 2023;52(4):1276–1285.

Pottegård A, Schmidt SAJ, Wallach-Kildemoes H, Sørensen HT, Hallas J, Schmidt M. Data Resource Profile: The Danish National Prescription Registry. Int J Epidemiol. 2017 Jun 1;46(3):798-798f.

Schmidt M, Schmidt SA, Sandegaard JL, Ehrenstein V, Pedersen L, Sørensen HT. The Danish National Patient Registry: a review of content, data quality, and research potential. Clin Epidemiol. 2015 Nov 17;7:449-90.

Wickham H. ggplot2: Elegant Graphics for Data Analysis. Springer-Verlag New York. 2016. https://ggplot2.tidyverse.org
